# Work for Disabled Soldiers

**Published:** 1921-07-16

**Authors:** 


### THE PUBLIC WELL-BEING.

### Work for Disabled Soldiers.

The rigid conditions of modern industry have added to the inevitable difficulties of what is admitted to be a national duty, the finding of suitable work for men disabled in the war. Where in olden days an employer would have taken back a man who had not strength to do a full day's work, and would have paid him according to his capacity, such a proceeding is now impossible in view of standardised wages and trade-union rules. Thus it befalls that many men, if they are to contribute to their own maintenance, must be trained to new work, and this is difficult for two reasons. In the first place, it is not easy for a man of mature years to take up new tasks. In the second, the work available is often not such as to appeal to the disabled. The magnificent altar frontal which was used at St. Paul's for the Peace celebrations was the work of soldiers in hospital, and proves that many men could do embroidery of both artistic and commercial value, but many of those who welcomed the work as a break in the monotony of convalescence look upon it as a woman's job, not fit for a man to earn his living by. And to others the making of toys seems a trivial occupation. Of course, it is not possible to take into account the prejudices of the men who are to be provided for, but, as far as possible, one would desire that their work should be of such utility that their self-respect should not suffer by their undertaking it.

From the Inter-Departmental Committee, over which Sir Montague Barlow has presided, much guidance may be expected upon this problem. It may be assumed that the colonies for tuberculous patients which have been established in healthy localities will be continued and increased. The men find useful and congenial occupation in market-gardening and other outdoor work. With modern means of transportation these colonies may be fairly distant from a town, and yet be commercially successful, and if worked on a co-operative basis the question of wages need not arise. Indeed, such colonies may be the means of infusing new life into some of our moribund villages. Hand-loom weaving, which has brought new life to many villages in remote corners of the land, might form a suitable trade for other centres. The instruction in poultry-farming given at St. Dunstan's is one of the marvels of our age, and in the War Seal Mansions for disabled men every encouragement is given to the occupants either to follow their former trade or take up a new one.

But with all that has been done an enormous amount remains to do. Money is wanted not only for the training of the men, but also for placing them in suitable conditions when they are trained. Many break down when they are set in ordinary industrial surroundings. And it is not always easy, or even possible, to place men after they have been trained. The fact that they cannot compete on equal terms with ordinary workers makes it all the more desirable that they should work in colonies, large or small, and for health's sake they are better in the country than in the towns. It is estimated that there are at present 100,000 partially disabled men trained for work. There is thus room for a large organisation. It is desirable that the work of the disabled men should as far as possible be employed on commodities which at present we get from foreigners. The successful diamond-cutting industry established at Brighton shows what can be done for our disabled soldiers without competing with other workers. But as this is a luxury trade it cannot absorb a large number of men. But investigation of this and other efforts at solving the problem will give valuable guidance to the Government Departments which are to solve it. These are the Ministry of Pensions, the Ministry of Health, the Ministry of Labour, and the Board of Agriculture. Intelligent co-operation between these should settle a question which otherwise must remain as a reproach to the nation.

